# 

**Published:** 1885-07

**Authors:** 


### THE BISTOURY:

Thad S. Up de Graff, M. D., Editor & Proprietor.

### CONTENTS FOR JULY, 1885.

**CONTRIBUTIONS.** PAGE.

The Gallivanters' Folly ........ BEN. H. PRATT ........ 131
Hats and Their Relation to Men ........ AUSBURN TOWNER ........ 133
Reality ........ J. B. CHANDLER ........ 136
A Neglected Profession ........ GEO. E. BLACKHAM, M. D ........ 138
Micrometers ........ PROF. D. R. FORD ........ 140

**MEDICAL ITEMS AND NEWS.**

Corn Cure—Catarrh Inhalant—Osgood's Cholagogue or celebrated Ague Cure—A Combination of Bromides for Epilepsy—Hair Tonic—Hemorrhoids—An Antiseptic Mouth Wash—Tonic Cough Mixture—Kennkle's Vegetable Worm Sprup—"Common Colds"—Haemoptysis—Toothache Cure—Eye Waters—Ticdouloureux—Cholera Infantum—Mixture for Plastic—Prespiration of the Feet—Hypodermic Solution—Anti-Hemorrhagic—Chronic Rheumatism—Epistaxis—Constipation and Dyspepsia—Mitral Murmur—Gastric Vertigo—Diarrhoea—Chorea—Fothergill's Formula for Asthma—Treatment of Bunions—Treatment of Ephelis or Freckles—Gonorrhoea—Amenorrhoea—Anemia—Dipsomania—Habitual Constipation (Prof. Bartholow)—Treatment of Furuncle Boil—Paper Towels for Surgical Purposes—Elimination of Phosphoric Acid in Insanity and Epilepsy—Nervous Headache—Acute Rheumatism—Lithiasis—Asthma—Diabetes—Pneumonia—Acute Bronchitis—Spermatorrhoea—Belladonna Poisoning ........ 142 to 146

**HOUSEHOLD HINTS AND RECIPES.**

Glacial Acetic Acid—Furniture Polish—A Harmless Soldering Mixture—To Render Fabric Uninflammable—Cheap and Good Cologne—Hair and Whisker Wax—To Preserve Eggs—A Paint for Blackboards—Bleaching Bones—Manufacture of Etching Ink—Chilblain Ointment—To Clean Hecktograph Pads—Lemonade Extract—Perfumed Oil for Pomades, Hair Oil, etc—Cleaning Mortars—To Clean Plaster-of-Paris Busts—Dammar Varnish—Perfume for Hair Oil and Pomades—Cleaning Rags—Liquid Solder for Tin Ware—Rubber Stamps—Artificially Colored Cigars—Bottle Glue—Substitute for Woman's Milk—Boroglyceride—To Remove Rust from Marble—Cement for Glass, Porcelain, etc—Plastic Clay for Suppositories .... 147 to 149

**EDITORIAL DEPARTMENT.**

A Typographical Error—"Bung Party"—Rink Hygiene—Our Friend—Temperance—Utility—The Baby Speaks—The Tennis—Great Britain—The War—A Well-known Elmira Clergyman—Advertising—Sea-Sickness—The Next Question—State Dam—Diagnosis of Discontent—Little Joe—Bermuda and its Results—Malaria—Cicada—The Tricycle—Blake ........ 151 to 154.

**TERMS.—50 Cents a Year Address the Bistoury, Elmira, N. Y.**

